# Provision of High-Quality Molasses Blocks to Improve Productivity and Address Greenhouse Gas Emissions from Smallholder Cattle and Buffalo: Studies from Lao PDR

**DOI:** 10.3390/ani12233319

**Published:** 2022-11-28

**Authors:** Peter Andrew Windsor, Julian Hill

**Affiliations:** 1 Sydney School of Veterinary Science, The University of Sydney, Camden, NSW 2570, Australia; 2 Ternes Scientific, Upwey, VIC 3158, Australia

**Keywords:** cattle, buffalo, greenhouse gas emissions, abatement, climate change management

## Abstract

**Simple Summary:**

Urgent responses to the climate change crisis are required, with concerns that cattle and buffalo are contributing to greenhouse gas emissions, particularly in developing countries where large ruminant production is inefficient. Recent studies in Lao PDR demonstrated that ad libitum supplementation of smallholder large ruminants with high-quality molasses nutrient blocks (MNB, 20 kg) with and without anthelmintics and 8% or 10% urea, provided from Australia (Four Seasons Pty Ltd., Brisbane, Qld, Australia), significantly improved productivity, average daily gains and milk production for MNB-supplemented animals compared to controls. ‘Emissions control molasses blocks (n = 200) were then formulated and distributed to beef farmers (n = 60) and two institutional farms to obtain block consumption rates (156 g/day) and farmer acceptance data. Modelling of greenhouse gas emissions (GHGe) intensity using Intergovernmental Panel on Climate Change (IPCC) Inventory software model V 2.69 of the recently published data on use of molasses nutrient blocks demonstrated a conservative net abatement of 350 kg CO_2_e over a 200-day feeding period, whereas modelling of the Emissions control molasses blocks identified an abatement of 470 kg CO_2_e per block consumed. We conclude that provision of high-quality molasses blocks to smallholder large ruminants may achieve impressive productivity gains and inclusion of greenhouse gas reducing agents improves the likely abatement of greenhouse gases during rumen fermentation.

**Abstract:**

Large ruminant production in developing countries is inefficient with low growth rates and likely high greenhouse gas emissions per unit of meat or milk produced. Trials conducted in Lao PDR from 2017 to 2020, studied ad libitum supplementation for 12 weeks with 20 kg high-quality molasses nutrient blocks (Four Seasons Pty Ltd., Brisbane, Australia), that were either non-medicated; fenbendazole-medicated (Panacur100^®^, Coopers Australia, 5 g/kg); triclabendazole-medicated (Fasinex^®^, Novartis Australia, 5 g/kg or 10 g/kg, respectively); or formulated with urea (8% or 10% urea, respectively). Average daily gains were determined for access to all molasses blocks and compared with access to control blocks, no supplementation, or previously determined free-grazing baseline average daily gains (55–84 g in cattle; 92–106 g in buffalo). Productivity was significantly improved following access to all molasses blocks. Average daily gains following access to 8% urea and control blocks were calculated for three age cohorts of cattle: young calves <8 m (238–298 g), growing cattle (143–214 g) and lactating cows (179–191 g). Modelling using IPCC Inventory software model V 2.69 of published data demonstrated a conservative net abatement of 350 kg CO_2_e was achievable over a 200-day feeding period. An additional trial of Emissions control blocks (n = 200) distributed to farmers (n = 60) and two educational institutions were conducted. Consumption rates (156 g/day) and farmer and institutional acceptance of these blocks were similar to our published findings with other molasses blocks, confirming all formulations of blocks improved animal productivity and body condition score, with healthier animals that were easier to manage. Modelling of changes in greenhouse gas emissions intensity identified an abatement of 470 kg CO_2_e per Emissions control blocks consumed, delivering a total project emissions abatement of 94 t CO_2_e. Provision of high-quality molasses blocks significantly improved smallholder large ruminant productivity and addition of greenhouse gas reducing agents is likely to achieve impressive abatement of greenhouse gas emissions due to improved efficiency of rumen fermentation and productivity.

## 1. Introduction

In developing agricultural systems, it is well recognised that both the concepts and practices of improved animal nutrition and health are slow to gain traction in many regions, with production losses regularly occurring. This is particularly the case in the tropical dry season, often when bovine parturition and lactation occur, and during the endemic and transboundary disease incursions that frequently occur in farming systems where biosecurity is absent [1,2,3]. These are major constraints that have been well documented and are currently remaining largely unmanaged [4,5,6]. In these developing country scenarios, identifying motivations for farmers to adopt sustainable practice changes is challenging. However, recently published studies from Lao PDR that documented the efficacy of a commercially available, farmer-applied high-quality molasses nutrient blocks (MNBs) strategy, suggests an increasing willingness and capacity of producers to address nutritional and health deficit concerns in Lao PDR livestock [4,5,6,7,8]. These recently published observations indicate that when efficacious MNB supplementation is incorporated into strategies to improve farm production, the dramatic impacts observed by livestock producers encourages their uptake of improved farm management, leading to increased livestock production efficiency and potentially, adoption of farm biosecurity [1,2,3,4,5,6,7,8]. This paper reviews these recent studies on the field use of MNBs for large ruminant production in Lao PDR and use of GHGe modelling that suggests that not only are MNBs capable of contributing to improved global production system efficiencies and food security, addition of greenhouse gas emissions (GHGe) reducing agents to the formulation, enables MNBs to potentially assist the change management required to address global climate crisis concerns.

## 2. Global Livestock Production and GHGe Issues

Livestock production accounts for approximately 40% of agricultural output in developed countries, with advances in genetics, pasture and feeding improvements, animal health prevention, plus other animal welfare and production management technologies, having reduced land requirements for livestock by about 20%, yet doubling meat production within the last 40 years [9]. Global meat and milk production has been projected to increase by another 19% and 33% by 2030, respectively [10], and contributing to feeding an estimated 8 billion people, with currently, over 80 billion animals slaughtered annually, producing 48,340 million tonnes of meat for human consumption [11]. To continue achieving these increasing demands requires substantial improvements in the adoption of existing and emerging ‘best practice’ husbandry, health, welfare and climate smart innovations for livestock production [12]. Improvements are required in feed resources, preventive health strategies and biosecurity, optimal manure management, food safe processing (e.g., risk-based meat safety assurance), animal and product marketing, and importantly, animal welfare and climate change adaption.

The global livestock sector is increasingly recognised as an important contributor to climate change though greenhouse gas emissions (GHGe) and particularly methane (CH_4_) and nitrous oxide (NO_2_) generated from rumen fermentation and manure management [9]. This is central to the UNFCCC activities for emissions abatement and adaptation of farming systems to a changing climate articulated through the Paris Agreement [13]. However, the Paris Agreement clearly identifies that abatement or climate adaptation must not result in reducing food production or food security. Thus, improving ruminant production efficiency and reducing CH_4_ and NO_2_ from fermentation is central to reducing GHGe from livestock production systems, achieving so-called emissions abatement via changes in emissions intensity aligned to strategies that abate rumen CH_4_ and NO_2_ directly. It has been estimated that improved production efficiencies could potentially assist the global livestock sector to reduce GHGs by as much as 30% [9]. However, achieving livestock production efficiencies and reduced GHGe per unit of meat or milk produced, will require both increasing improvements in nutritional management and more effective strategies for managing the impacts of increasing climate variability, including preparedness for droughts, fires, storms, floods and other environmental impacts [14].

GHGe from livestock are estimated at 7.1 gigatonnes CO_2_-eq per annum, representing 14.5% of all human-induced emissions. The animal-sourced foods (ASF) commodities contributing most GHGe from livestock are those from cattle production systems, including estimates of 4.6 gigatonnes CO_2_-eq or 65% of sector emissions from beef and cattle milk production. Beef contributes 2.9 gigatonnes CO_2_-eq, or 41%, and cattle milk 1.4 gigatonnes CO_2_-eq, or 20% of total sector emissions, respectively. Buffalo milk and meat contribute 0.6 gigatonnes CO_2_-eq or 8%, with small ruminant milk and meat contributing the balance of 6% [2,6]. Beef produced by dairy cattle has generally lower emission intensity than beef produced by specialised beef cattle, as GHGe from reproductive animals are allocated to milk and meat in the case of the dairy herd, and to meat only in the case of the beef herd. The allocation of individual GHG sources to the estimates of emissions, reported as CO_2_-equivalents (CO_2_e) is important. The majority of GHGe (>80%) from smallholder ruminant livestock systems can be ascribed to methane (CH4). The signing of the Global Methane Pledge by 130 national signatories at Cop26 aims to achieve global action for existing international CH_4_ emission reduction initiatives. It promotes advancing of technical and policy work that supports voluntary domestic actions of signatory participants [15], contributing to a collective effort to reduce global CH_4_ emissions by at least 30% from 2020 levels by 2030. Participants also committed to moving towards using the highest tier IPCC good practice inventory methodologies that are continuously working to improve the accuracy, transparency, consistency, comparability, and completeness of national greenhouse gas inventory reporting under the UNFCCC and Paris Agreement [13,15].

A number of different strategies to mitigate GHGe in the livestock sector have been explored, classified according to whether the mitigation achieved is derived from:

(i) reducing total emissions by inhibiting CH_4_ production in the rumen; or
(ii) reducing emissions intensity (Ei) by reducing CH_4_ per output unit without directly targeting methanogenesis [14].

Those strategies classified as directly targeting rumen methanogensis include chemical inhibitors, electron acceptors (e.g., nitrates), ionophores (e.g., Monensin), dietary lipids and a range of plant bio-actives generally configured similar to isoprenoids. Strategies that reduce Ei include increasing the digestibility of the dietary intake; improving preventative health and welfare management to reduce disease burdens; increasing reproductive performance; and selective breeding programs that result in higher productivity. Whilst increasing production efficiency of ruminants offers promise as a means of reducing the carbon footprint from livestock production, there are concerns that this could compromise animal welfare. Research exploring strategies that reduce GHGe should simultaneously aim to improve animal welfare [14,16] and both nutritional supplementation and improved health prevention and management strategies, are required.

## 3. Inefficiency of Smallholder Livestock Production Systems

Of importance to considerations of the global carbon footprint of livestock production, is the inefficiency of developing country agriculture, representing only about 20% of agricultural output and dominated by smallholder farmers, producing over 30% of the global food supply [17,18]. Ruminant systems in developing countries are considered resource-use inefficient, with high yield gaps in most of these production systems [19]. Increasing the efficiency of the livestock sector through sustainable intensification practices presents an opportunity for research and development to provide more sustainable solutions for the 17 billion animals in the world eating, excreting and using substantial amounts of natural resources, mostly in the developing world, to contribute to feeding the estimated >9 billion people by 2050 [10]. Achieving this requires that production systems become market-orientated, better regulated, and more socially acceptable so the right mix of incentives enables these systems to intensify, whilst minimising the inevitable increases in zoonotic, food-borne, transboundary and other emerging diseases.

In South-east Asia, smallholder livestock production is in transition yet remains inefficient, with this situation persisting, despite the rapidly rising demand for milk and meat in countries where historically there has been very limited access to protein-rich ASFs [9]. In the Greater Mekong Subregion (GMS) countries (including southern China, Myanmar, Lao PDR, Thailand, Cambodia and Vietnam), large ruminants are decreasingly required for their historical role as draft animals and stores of livelihood wealth. However, their contribution to production of beef and milk is particularly inefficient, incurring high emissions of GHGe. These high emission intensities are due to low feed digestibility, less efficient herd management practices and very low reproduction performance [1,2,3,9,18]. Interventions that significant increase the efficiency of large ruminant livestock production in developing countries may potentially offer very important opportunities for mitigation of GHGe from the livestock sector, potentially enabling the global livestock sector to reduce GHGe by as much as 30% [9,18]. A multi-intervention livestock development strategy involving a combination of nutritional and health interventions has been proposed for scale-out to assist smallholder large ruminant livestock farming efficiency in developing countries [1,2,8]. The strategy includes a combination of establishing forage plantations and improvements in feeding systems, with multiple health interventions involving efficacious vaccination, biosecurity, and parasite management programs. Within this strategy, a nutritional gap has been identified where forage digestibility and availability falls to such an extent that animal productivity falls to maintenance or even sub-maintenance levels. Strategic supplementation has been demonstrated to assist in preventing low animal efficiency. In particular, a strategy that uses MNBs provided ad libitum to improve rumen function and enhance large ruminant productivity, has resulted in increasing lactation yields [1,2].

## 4. Use of Molasses Blocks as a Large Ruminant Supplement

Use of MNBs has enabled strategic supplementation of the generally inefficient large ruminant production system in a number of countries [19,20,21]. The technology is simple to deploy in field and is widely accepted by farmers [19]. However, variations in the content and the quality of blocks used in various locations have created variable results and raised concerns of the validity of this approach [22,23]. Recent experience has found that the key to effective supplementation with MNBs is the establishment of a robust manufacturing method that yields a consistently produced, high-quality block that is suitable for tropical conditions. Sufficient hardness in the block is necessary to assist in controlling consumption, ensuring contents are ingested at a consistent rate [1,2]. The MNBs also need to be deployed appropriately to strategically address the various nutritional or animal health constraints through a self-feeding production system [24]. MNBs enable nutritional supplementation, particularly during the dry season when feed quality is low, reducing the rate of decline in body condition score [1,2]. MNBs may also enable delivery of medication for control of endoparasitism where facilities for direct administration are absent [20,21]. The published trials undertaken by this research group in Lao PDR have provided robust evidence that the constraints of using molasses blocks can be managed with the strategic use of a range of high-quality MNBs. These MNBs have achieved very high rates of acceptance by farmers that reflects the impact of nutritional supplementation and strategic use of medication on individual animal and herd health performance in smallholder large ruminant farms [1,2,8,20,21].

The successful use of high-quality MNBs in smallholder large ruminant tropical systems was initially driven by a need to address the challenge of achieving sustainable control of helminths in beef animals in Lao PDR [25]. The use of MNBs to deliver important animal health medication was initially aimed at both improving the nutrition of the animal and addressing endoparasite control. Trials involved incorporation of either fenbendazole in the feed block where (i) helminths and in particular, *Toxocara vitulorum,* were limiting survival and growth rates of young beef animals [25]; or (ii) triclabendazole where *Fasciola gigantica* was limiting productivity of adult animals and compromising the integrity of high value hepatic tissue at slaughter [26]. Successful results with MNBs containing an anthelmintic for endoparasite control, encouraged subsequent trials with MNBs that focused on nutritional supplementation in the late dry season, particularly for lactating cows, with and without the inclusion of 8% urea in the block. This intervention also demonstrated considerable improvements in growth rates and weight gains in different age cohorts, in the order of 2.5 to 5 times average daily gains (ADGs) compared to baseline longitudinal data collected from animals not provided with MNB supplements [1]. As the most impressive ADGs occurred when animals accessing MNBs were under 8 months of age, it was suggested that this was most likely due to improved lactations of their dams. A subsequent trial in lactating Asiatic swamp buffalo used for milk production and provided with ad libitum supplementation with MNB containing 10% urea, confirmed that up to 31% improvement in milk production may occur [2]. The economic potential of this intervention was supported by partial budget analysis and suggested that because of the considerable socioeconomic benefits and acceptance by farmers of high-quality MNBs, commercial opportunities for provision of the technology into smallholder large ruminant production systems may accrue.

Although the research trials suggested there was considerable potential for MNBs to create positive improvements in rural livelihoods for poor rural families through progressing livestock production in developing countries, it was evident that MNBs could also potentially reduce the GHGe footprint of large ruminant production. Our hypothesis considered that MNBs could deliver both an increase in efficiency of large ruminant productivity and diminish the Ei of production. When combined with preventative health (e.g., vaccination programs) and other nutritional improvement strategies (e.g., forages) and delivered in a co-learning participatory environment with smallholder farming families, it is expected that substantial and sustainable improvements in livestock production efficiency can accrue. Importantly, as improved large ruminant productivity is considered to have the potential to enable the global livestock sector to reduce GHGe by as much as 30% [9] and help address the risks of the impending climate change catastrophe, further assessment of the GHGe abatement potential of MNBs in smallholder large ruminant livestock systems is required, particularly as ‘scale-out’ of this multi-intervention co-learning strategy has been proposed [1,2,8].

The major constraints to large ruminant production in Lao PDR have been well documented and remain largely unmanaged, despite increasing research suggesting that appropriate interventions can substantially ameliorate these losses [1,25,26,27,28,29]. These recently published studies have documented the efficacy of a commercially available, farmer-applied MNB strategy, suggesting an increasing willingness and capacity of producers to address nutritional and health deficit concerns in Lao PDR livestock, provided products are available that motivate farmer understanding of how to improve animal productivity, health and in particular, biosecurity change management [1,4,5,6,7,8]. These recently published observations indicate that when efficacious MNB supplementation is incorporated into strategies to improve farm production, the dramatic impacts observed by livestock producers encourages their uptake of improved farm management, leading to increased livestock production efficiency and potentially adoption of farm biosecurity [1,2,3,4,5,6,7,8,20,21]. These studies on the field use of MNBs in Lao PDR suggest they are capable of both contributing to improved global production system efficiencies, food security, and may potentially address global climate crisis concerns.

## 5. Published Studies on a Range of MNBs

The studies in Lao PDR were initiated to address the challenge of sustainably controlling *T. vitulorum* burdens in calves, despite the increasing availability of the recommended oral treatment with the anthelmintic pyrantel [20,25]. It was identified that on many smallholder farms, achieving pyrantel administration during the narrow range of efficacy of 10–16 days of age was difficult. This reflected the random calving periods due to absence of controlled breeding management, with cows often calving remotely. Further, farmers often lacked skills in animal handling and had poor knowledge of ageing of calves accurately, the identification of relevant clinical signs of endoparasitism, and the most appropriate and efficacious therapies available. Further, the widespread absence of herd management infrastructure to restrain and identify animals, the isolation of villages from government-supported animal-health services reducing regular access to veterinary treatments and expertise, and the low-level resources of government livestock extension services, all contributed to the continuation of complacency and lack of motivation to address the current levels of low animal productivity in the Lao PDR subsistence large ruminant livestock systems. To successfully address these challenges, it was determined that motivation of farmers required the use of interventions that readily produced visible improvements in clinical signs, body condition scores and animal values. As nutritional and health deficits both contribute to low productivity levels, an intervention that addresses both deficits is required. The use of high-quality MNBs that enables nutritional supplementation and also provides the option of ease of administration of oral anthelmintics, was considered as a potentially important intervention to motivate farmers to transition from subsistence livestock systems [20,21].

MNBs provide digestible energy, protein (and/or non-protein nitrogen), minerals and vitamins to rice-straw and native-grass diets, where they are lacking. This is facilitated by highly palatable molasses, a high-energy supplement that increases the efficiency of rumen microbial activity by providing readily available energy for rumen microbial growth and synthesis of microbial protein [1]. The resulting enhancements to ruminant productivity can be further increased where endoparasitism is a production-limiting constraint, by addition of efficacious anti-parasitic medication. The anthelmintics fenbendazole (FBZ) or triclabendazole (TBZ) were included in MNB formulations, potentially providing nematode or trematode control, respectively, through parasite suppression or death [20,21]. These initial studies did not include urea (a source of non-protein nitrogen) in block formulations to enable the focus of the trials on the assessment of MNBs on the effects of nutritional supplementation with or without an anthelmintic, on growth rates and parasite burdens. Urea is often added to molasses blocks to further improve the digestibility and intake of roughages by safely providing a soluble and rapidly degradable source of nitrogen that is hydrolysed in the rumen to yield ammonia. As the addition of nitrogen as urea in MNBs enhances microbial degradation of dry matter and synthesis of protein, studies examining addition of 8% and 10% urea to the blocks were also undertaken [1,8].

MNBs used in all the trials were manufactured in Australia (Four Seasons Pty Ltd., Brisbane, Qld, Australia) and transported to Lao PDR for distribution to farmers owning large ruminant livestock in various, mostly northern provinces. The constituent ingredients of the blocks were initially as described [20,26] and the components progressively adjusted to ensure they: resisted tropical degradation from heat, humidity and rain; delivered components safely; enabled sufficient intake of nutrients to manage deficits including optimal availability of phosphorus, sulphur, nitrogen, and other minerals and vitamins; safely provided levels of anthelmintics (for parasite control blocks only), and urea (for dry-season and lactational enhancement blocks only) that could achieve efficacious levels of parasite suppression or elimination, and enhanced rumen microbial protein synthesis, respectively. Further modifications of the MNB contents including GHGe reducing agents (including oils, ionophores etc.) then led to the development of ‘Emissions control blocks’ (EMB) for delivery and evaluation on farms in Lao PDR (discussed below).

The MNBs weighed 20 kg, measured 400 mm × 180 mm × 260 mm, and were distributed to households at a rate of one block/20 large ruminants. Farmers were instructed to place blocks on elevated stands under shelter or in animal houses, then request new blocks when the previous blocks were consumed, enabling consumption rates to be determined. Due to unrestricted animal movement in smallholder farms, adult and juvenile bovids had access to the blocks, limiting calculations of calf consumption to estimates. Animals were maintained under normal field management conditions, typically involving free-grazing of grasses on roadsides, paddy-lines or nearby forested areas during the day, with containment in animal housing at night where they had unrestricted access to blocks. Farmers were advised to provide ad libitum water.

### 5.1. Baseline Data and Use of Non-Medicated Blocks for Nutritional Supplementation (NMB)

The low-level large ruminant livestock production parameters in Lao PDR were determined in a series of longitudinal studies conducted prior to the MNBs studies reviewed here [4,5,6,7]. Importantly, a 3-year longitudinal study involving 1500 head of cattle and buffalo from the three northern provinces of Luang Prabang (LPB), Xiengkhouang and Huaphan, that were ear-tagged and weighed every 3–4 months between 2008 and 2011, produced 10 data-collection points and enabled baseline production variables, including live weight, average daily weight gain (ADG) and reproductive performance, to be obtained [4,5,6]. The studies aimed to evaluate the impact of the introduction of forages, health and other interventions into the production system. Although significant differences in ADG of cattle between provinces (*p* < 0.001) was observed, the data identified that the northern Lao PDR free-grazing production system was characterised by very low predicted mean weights and ADGs. Further, significant seasonal fluctuations in these measured performance parameters were observed, reflecting limited feed availability in the dry season from December to May. Although the project introduced forage plantations in targeted villages, there was variable uptake of this intervention. Only one of three provinces was successful in adopting this practice, with over 50 ha of forages grown in LPB by project completion in 2012, explaining the higher ADG in cattle in this province than at other sites. The cattle mean ADG of 84 g in LPB was significantly higher than the ADGs from 54 g observed in the other two provinces. There was no significant difference in buffalo predicted mean ADG among the provinces (*p* = 0.05) with an overall predicted mean ADG in LPB of 106 g compared to 92 g in the other provinces. Of interest was that in a similar longitudinal study in southern Cambodia, cattle in villages where forages were successfully established had a significantly higher ADG of 116 g, compared to 49 g during the 4-year study. The different outcomes of two similar projects conducted in neighbouring countries were considered mostly attributable to differences in the uptake of forage plantations between the sites [27].

The need to provide a more seasonally balanced supply of nutrients and improved farm management to progress Lao PDR cattle and buffalo productivity is well recognised, with forage plantations promoted to assist management of feed shortages and provide a resource for fattening of large ruminants before sale to increase animal values. An on-farm fattening trial demonstrated that cattle and buffalo in fattening stalls (320 g and 217 g) had significantly greater ADG than those free-grazing (40 g and 85 g), respectively [4,5,6]. The challenge involved in providing adequate knowledge on the planting, care and harvesting of forages, the level of investment required, plus the improved husbandry and basic biosecurity practices that are necessary to protect the increased investment, have also been recognised as has the option of investigating other methods of improving nutritional and feeding management [4,5,6]. The use of MNBs has been suggested as a convenient means of more rapidly progressing this requirement. Whilst the proposed strategy of promoting a combination of established forage plantations and improved feeding systems (e.g., stall fattening), with multiple health interventions involving efficacious vaccination, biosecurity, and parasite management programs, recent findings have indicated that this strategy can be precipitated by use of high-quality MNBs to improve rumen functionality [1,2].

In the majority of the trials discussed below, comparisons were made between ‘medicated’ and ‘non-medicated’ (NMB or so-called ‘control blocks’) to test the efficacy of the addition of either anthelmintics or/and urea to the block. It was clear that in all trials, due to the poor underlying nutrition of cattle and in particular, suckling calves, the NMB ‘control blocks’ were effective in providing nutritional supplementation and on occasions, appeared to more effectively lower the prevalence of endoparasitism and increase the ADG (Table 1) in calves than blocks containing an anthelmintic. The benefits of feeding unmedicated MNBs in the dry season when nutritional availability is the lowest can result in significantly increased milk yield, dietary intake, weight, and significantly reduced postpartum anoestrus in lactating indigenous cattle [1,2].

### 5.2. Fenbendazole-Medicated Blocks for Helminth Control (FMB5)

In Lao PDR, cattle and buffalo are mostly held by smallholder farmers in traditional low-input subsistence systems with low reproductive efficiency and poor calf survival, with rates of annual calf morbidity and mortality of 42.6% and 37.3%, respectively, reported in 2010 [25]. These high rates of calf losses are most often attributed, at least in-part, to the gastrointestinal nematode parasite *Toxocara vitulorum* that affects cattle and buffalo calves less than 3 months of age and occurs widely in bovid populations in Lao PDR [25]. Field trials were conducted to provide efficacy data on an alternative, easy-to-use anthelmintic treatment, by provision of 20 kg MNBs, either medicated with fenbendazole 5 g/kg (FMB5; Panacur100^®^, Coopers, Australia) or non-medicated (NMB). Participating villages were randomly allocated to the following treatments: (i) conventional orally administered pyrantel; (ii) access to FMB5s; (iii) access to NMBs; and (iv) no access to blocks (negative control). Faecal egg counts per gram of faeces (FEC) were assessed by the flotation method to provide eggs per gram (EPG) data and weights were regularly monitored in cattle (n = 171) and buffalo calves (n = 44) under field conditions for 48–56 days.

Results identified that NMB treatment was associated with the fastest reduction in predicted average EPG at 2% per day, with FMB5 and pyrantel having an equivalent reduction of 1% per day, relative to the negative control (*p* = 0.062). Predicted average weight also differed significantly among treatments, with pyrantel and NMB having the greatest ADG at 220 g and 216 g respectively, with FMB5 at 200 g; all were higher than for control calves at 170 g (*p* = 0.002) (Table 1). In buffalo calves, treatment was not significantly associated with FEC or weight. A subsequent trial corroborated that FMB5 and NMB treatments were associated with increasing FEC reductions in cattle at 3% per day, relative to control calves (*p* = 0.007). Again, the NMB treatment had the greatest predicted ADG at 200 g, compared with FMB5 calves at 160 g and control calves at 150 g (*p* = 0.005). It was concluded that both FMB5 and NMB have potential applications in reducing environmental contamination of *T. vitulorum* eggs and may improve calf growth in low-input systems, although controlling for variability in calf weight and *T. vitulorum* burdens to optimise anthelmintic doses in the block formulation was advised [20].

### 5.3. Triclabendazole-Medicated Blocks for Trematode Control (TMB5, TMB10)

*Fasciola gigantica* is an endemic parasite in smallholder cattle and buffalo production in many tropical developing countries, especially where facilities for annual or strategic anti-trematode treatments are absent. Surveys for liver fluke in Lao PDR found that 73.3% of villages from five northern provinces had at least one faecal egg count-positive animal, and slaughterhouse surveys of bovid livers (n = 123) identified that 70.7% had gross hepatobiliary lesions consistent with *F. gigantica* infection [28,29]. A study examined the potential for sub-therapeutic doses of triclabendazole provided in medicated molasses blocks offered to large ruminants, to suppress Fasciolosis [21]. The trial involved cattle (n = 241) allocated into three groups: (i) triclabendazole (as Fasinex^®^, Novartis Animal Health Australia, Pty Ltd., Maquarie Park, NSW, Australia) medicated molasses blocks (TMB5) with each tonne of blocks containing 0.5 kg triclabendazole; (ii) non-medicated molasses blocks (NMB); and (iii) a control group (no treatment). Data and faecal samples were obtained at Weeks 1, 4, 8 and 12 for FEC determination by the sedimentation method. Reductions in trematode FEC in the TMB5 group of 90.48% and a mean FEC of 4 +/− 17 eggs per gram of faeces at 12 weeks post-treatment was observed, with liveweight increasing from 174.60 (3.35) kg to 191.50 (3.69) kg in Weeks 1 and 12, respectively (*p* = 0.001) providing an ADG of 201 g. Reduction in FEC in the NMB group was also observed, by 28.78% and 18.96%, with liveweight increasing from 179.50 (3.35) kg to 189.90 (6.05) kg in Weeks 1 and 12 respectively (*p* = 0.3), providing an ADG of 124 g (Table 1). The study suggested that productivity was enhanced when triclabendazole was added to the blocks, delivering parasite suppression or potentially therapeutic doses on ad libitum feeding of TMB5 [21].

An additional study was then conducted to confirm the therapeutic potential of using the recommended dose of triclabendazole (Fasinex^®^, Novartis Australia, Maquarie Park, NSW, Australia) with each tonne of blocks containing 1.0 kg triclabendazole (TMB10). As the provision of TMB10 led to elimination of the trematode FEC within several weeks of access to the blocks, the TMB10 were replaced by NMB within a month of commencement of the trial. It was concluded that these findings may offer a convenient trematode parasite management and nutritional supplementation strategy for smallholder farmers. This is particularly important in Lao PDR (and some other countries) where unmanaged *Fasciola* spp. infestations reduce ruminant productivity, facilities for animal restraint to enable delivery of oral anthelmintics are largely non-existent and importantly, farmer knowledge is poor. A survey of farmers (n = 326) on knowledge of liver fluke and its management in their large ruminants, identifying 93.1% of farmers had no knowledge and 6.9% minimal knowledge of the parasite and impacts on large ruminant production [29].

### 5.4. Urea Blocks for Improving Lactation Yields (UMB8, UMB10)

The impact of ad libitum supplementation of cattle with high-quality MNBs (20 kg) containing either 8% urea (UMB8) or nil urea (NMB), was examined in field trials in the late dry season on smallholder farms in northern Lao PDR [1]. The trials compared weight changes and ADG data of young calves <8 months of age (n = 25); growing calves 8–24 months (n = 35) and lactating cows (n = 46), of the indigenous breed when accessing either UMB8 or NMB, with data collected at Weeks 1, 4, 8 and 12. Results indicated that smallholder farming cattle accessing UMB8 were heavier than those accessing NMBs at every collection day and in young calves these differences were statistically significant (*p* < 0.05). ADGs were also higher in cattle accessing UMB8 than in those accessing NMBs, with young calves having the highest ADG, followed by growing calves and lactating cows (Table 1), although differences in ADG between UMB8 and NMB cohorts were not considered significant (young calves *p* = 0.562; growing calves *p* = 0.509; and lactating cows *p* = 0.993). A survey of the participating farmers identified that they considered that the blocks contributed greatly to herd management and improved sale-ability of their cattle.

**Table 1:** Summary of mean ADGs (g/day) achieved in molasses blocks studies in Lao PDR.

Study	Cattle	Buffalo	Reference
Baseline	54—84	92—106	[4,5,6]
FMB5 *	200	212	[20]
NMB #	216	232	[20]
TMB5 **	201	na	[21]
NMB #	124	na	[21]
UMB8 calves < 8 m	265	na	[1]
NMB calves < 8 m	261	na	[1]
UMB8 growers	237	na	[1]
NMB growers	198	na	[1]
UMB8 lactating cows	190	na	[1]
NMB lactating cows	187	na	[1]
UMB8 research cows	229	na	[1]
NMB research cows	236	na	[1]

* FMB5:fenbendazole 5 g/kg block; # NMB: non-medicated block; ** TMB5: triclabendazole 5 g/kg; UMB: 8% urea block.

A pen study was also conducted at a research station involving mature, lactating crossbred cows (n = 37), comparing growth data from accessing UMB8 compared to NMBs. Results identified that ADGs in these pen trials were not significantly different (*p* = 0.933) between the lactating cows accessing UMB8 or NMBs (Table 1). Surveys of farmers using blocks confirmed that their animals were calmer and healthier, had better coat condition with minimal external parasites, and these farmers wished to purchase the blocks. It was concluded that provision of UMB8 and NMBs in the late dry season improved cattle growth rates that were far superior to the base-line growth data obtained from similar cattle on similar farms in Lao PDR [1].

**Figure 1:**
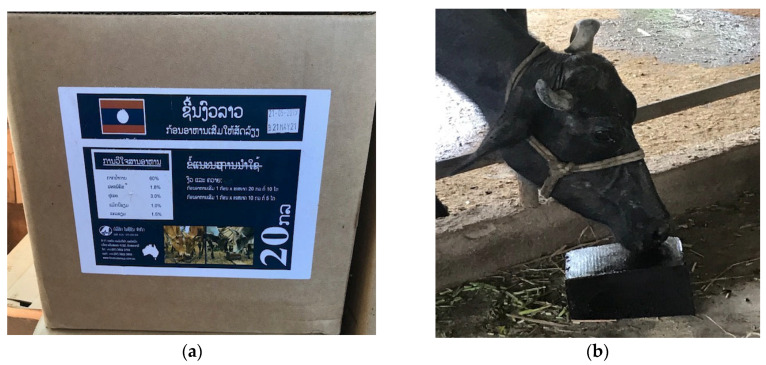
(**a**) MNB provided to farmers in Lao PDR; (**b**). buffalo cow consuming UMB10.

As the UMB8 study found the highest ADG was in young calves (<8 m age), this was considered most likely attributable to enhanced lactation yields of their dams. To investigate this, a trial in a recently established commercial Lao Buffalo Dairy (LBD), examined dietary supplementation of lactating buffalo cows with molasses blocks containing 10% urea (UMB10). The trial involved three groups of nine buffalo cows in mid-lactation that were randomly selected, with two groups receiving ad libitum access to UMB10 (Figure 1a,b), with the remaining group free of block supplements [2]. All animals were daily fed fresh Napier grass (30 kg), corn (750 gm), rice bran (1.45 kg), plus accessed fresh Mulatto grass. Daily milk production (DMP) and body condition score were recorded for the 2 months of access to UMB10. Average DMP for the two supplemented groups were 1.02 L and 0.96 L, compared to 0.78 L for the control group. This suggested improved milk productivity of 31% and 24% for the two groups accessing UMB10. Partial budget analysis identified a strong incentive for use of the molasses blocks, with a net profit of USD408 and USD295 over a 30-day period for the supplemented groups [2].

## 6. Evaluation of Emissions Control Blocks for Increased Abatement of GHGe (EMB)

### 6.1. Background and REVIEW of Data from Published Studies

The current practices of dry season feeding in Lao PDR impact negatively on large ruminant body weights, especially in lactating females. Longitudinal data demonstrating that in the late dry season, ADGs of −9 to 71 g in cattle and −21 to 23 g in buffalo were recorded and were considerably lower than early wet season ADGs of 208–212 g in cattle and 223–282 g in buffalo [1]. As the early dry season also coincides with both the calving period and increasing demand for animal sales for post-harvest festivities in Lao PDR, decreased animal condition in the dry season has negative implications for on-farm productivity and profitability, with poor quality dry season feed availability compromising lactation performance, extending post-partum anoestrus and reducing livestock sale values. As Lao PDR smallholder farmers use unrestricted mating, uncastrated males are common and managed weaning is rarely practiced, extended inter-calving intervals have been recorded, estimated at 14–20 months in cattle and 19–26 months in buffalo [3,4,5,6].

These constraints to forage availability indicate that additional strategies to provide nutritional supplementation of large ruminants are required. MNBs were suggested as offering a more rapid project intervention ‘entry point’ to enhance large ruminant production efficiency in smallholder systems [1]. MNB supplementation of animals for 84 days with high-quality MNBs, manufactured and delivered from Australia (Four Seasons Pty Ltd., Brisbane, Qld, Australia) provided energy and mineral supplementation for grazing ruminants. Inclusion of urea in the dry season was used to provide non-protein nitrogen, increasing feed conversion efficiency through amino acid and protein synthesis during digestion from enhanced microbial growth, leading to improved utilisation of roughages [30,31]. Reviewing the recently published data on the outcomes of feeding of various MNBs to beef cattle and dairy buffalo in Lao PDR, provides an opportunity to consider the potential impacts of these interventions on GHGe via productivity and Ei improvements, comparing these outcomes with those from more recently designed MNBs (EMBs) targeting abatement of GHGe.

### 6.2. GHGe Abatement Study from Provision of EMBs

MNBs (n = 200) weighing ~20 kg each, designated as ‘Emissions control blocks’ (EMBs) were shipped to Lao PDR from Brisbane (Four Seasons Company Pty Ltd., Brisbane, Australia) for a logistics trial conducted in villages and two research farm in LPB province. The composition of the EMBs included cane molasses, urea, canola meal, salt and a blend of macronutrients and micro minerals. The block supplements were formulated to resist meltdown from tropical heat, humidity and rain; deliver components in a safe preparation enabling optimal intake of nutrients, including readily available phosphorus, sulphur, nitrogen, and other minerals; and contain GHGe-reducing agents. Animals had ad libitum access to native pastures in daylight hours. No additional supplementation was provided and no disease issues of relevance were observed during the trials. The EMBs were distributed to farmers (n = 60) accompanied by a recording form for completion by participating farmers that was signed on receipt of the blocks. Concurrent feeding trials to validate EMB consumption and ADG from access to EMBs, was also conducted at the Northern Agriculture and Forestry College (NAFC) and Souphanouvong University (SPU) farms in LPB Province, Lao PDR, using protocols developed in previously published trials [1,2,20,21]. Modelling of biological responses and GHGe, including changes in Ei, was conducted, then reviewed by the Ministry of Agriculture and Forestry, Department of Livestock and Fisheries (DLF) in Lao PDR, with certification of emissions avoidance issued by the DLF.

### 6.3. Participatory Villages and Other Agencies

Participating villages receiving EMBs were located in LPB province in Lao PDR and included farmers in: Jok Village, Kok mun Village, Meuang Khaiy Village, Nar ngiw Village, Nong bouakham Village, Nong Jong Village, Ou Village, Som Village, Sungkhalok Village, Thim som Village, Thinkeo Village, with EMBs also used at the NAFC and SPU. Following delivery of EMBs to the smallholder large ruminant farmers across the 11 villages, feedback from farmers was obtained. These reports confirmed that feeding the blocks to large ruminants increased animal productivity, with improvements in body condition, with improved feed intake and animals had shinier hair coats and were more easily managed. Farmers appreciated that the cattle returned more readily of an evening to the overnight animal housing sheds, with participants agreeing that animal performance had increased, reflecting increases in the values of animal. It was agreed that yields of meat and milk for local communities had increased by access to EMBs, with a number of villages adopting milking of buffalo following participation in training programs provided by the LBD. No issues or health problems were noted with the feeding of the EMBs.

### 6.4. Measurement of EMB Consumption

EMBs provided for measurement of consumption rates, were available ad libitum to growing buffalo (n = 10; ~300 kg LW) managed conventionally on pasture, with weight of blocks and days until each block was consumed, recorded at SPU. Four feeding trials occurred and it was determined that the average rate of block consumption was 156 g/day (n = 1160 cow days). Intakes of the MNBs were comparable to previously published feeding trials in Lao PDR [1,2,20,21] and Australia [24]. Animal performance was estimated as 0.23 kg ADG. Feed intake for these animals was estimated as 2% of body weight.

## 7. Modelling of GHGe Abatement of MNB and EMB Studies

High-level modelling using IPCC Inventory software model V 2.69 was conducted on the published data from Lao PDR of buffalo fed ad libitum MNBs with 10% urea [2]. The conservative net abatement was used to calculate an aggregated of kg CO_2_e over a 200-day feeding period across the entire Lao PDR buffalo herd of approximately 775,000 head. IPCC Inventory V 2.69 relies on the calculation of GHGe on an individual animal basis and was extrapolated to herd scale emissions. This enabled a series of baseline emissions calculations that represented a ‘business as usual’ model; i.e., non ’additionality’ in animals not receiving MNBs. The interpolation of data collected from the field trials at SPU, provided information to calculate ‘project’ emissions; i.e., animals receiving MNB’ as EMBs. Project emissions represent a case where the EMBs is additional to the ‘business as usual’ feeding system and therefore allows the calculation of the difference of herd Ei (kg GHGe/kg ADG) between project and baseline models. This approach to Ei calculations for non-lactating buffalo is identical to that taken for lactating cattle using published resources [32,33,34,35,36,37,38].

For Lao PDR, the calculation of the abatement was based on the following formula:

1. Consumption of feed per day x abatement per kg = kg abatement/day (Project abatement)
2. Abatement per kg of supplement = kg abatement per kg supplement
3. Methane production (kg CH_4_/day) calculated for the baseline animals
4. Methane production (kg CH_4_/day) calculated for project animals (baseline abatement/day)
5. WP × kg CH_4_ /day = kg CO_2_e /day
6. Days fed × kg CO_2_e/day = total emissions abated.

Abatement per kg supplement was calculated in accordance with the concentration of known GHGe mitigation compounds and the impact of those compounds on the measured reduction in rumen CH_4_ production. The method requires declaration of the feed block composition and the measured abatement. The abatement in rumen CH_4_ emissions must be established through a meta-analysis of at least three peer-reviewed publications in reputable journals that are listed in the Science Citation Index Expanded. The efficacy of feed additives is influenced by dose, diet, production system, type of animal, and random variation. Therefore, a meta-analysis that considers these factors is necessary to obtain efficacy estimates within the range of the data used for the meta-analysis. The modelling using IPCC Inventory software model V 2.69 of the published data on use of UMB10 demonstrated that a conservative net abatement of 350 kg CO_2_e was achievable over a 200-day feeding period. In Lao PDR, the buffalo herd size is approximately 775,000 head, providing a potential total in-country abatement of 271,250 t CO_2_e per annum. As the average herd is about six animals, an individual producer system may only abate <1 tonne CO_2_e per annum. Hence an aggregation model has been designed and whilst there are verifiable examples of where the yield of milk is higher than the buffalo identified as unselected for milk production [2], the findings suggest that the net abatement achievable from use of EMBs is likely to be greater than the volumes reported as:
Emissions (baseline) per head1977 kg CO_2_e per annumEmissions (project) per head1182 kg CO_2_e per annumEmissions abatement (per block consumed)470 kg CO_2_e per blockTotal project emissions abatement94 t CO_2_e

## 8. Sustainable Development Goals (SDGs)

Consideration of the project alignment with SDGs was undertaken using the approaches outlined by United Nations Development Program [39]. Discussions with staff from the Lao PDR DLF identified alignment of the study with the four SDGs as central to the project, including: 1 (No poverty), 2 (Zero hunger), 13 (Climate action) and 17 (Partnerships). SDG contributions of future projects could be monitored through: (i) the net abatement of emissions (SDG 13); (ii) increases in supply of milk and milk products, and meat or other co-products (SDG 1, 2) as a result of the strategic supplementation (SDG 17); and (iii) tracking the response of smallholder farming systems to the benchmarks determined by UNDP and the individual nominated SDG, other than SDG 13 (Climate change).

## 9. Discussion

Molasses blocks have been advocated for delivery of improved nutrition for ruminants for many years, particularly in developing countries [19]. These recent studies in Lao PDR have confirmed that high-quality molasses blocks provide a robust means of delivering nutritional and health interventions that substantially boost productivity, with the options of addition of anthelmintics for endoparasite control, and/or urea for improved rumen fermentation, and potentially, GHGe reducing agents to reduce the carbon footprint of large ruminant livestock production. The initial trials examined the control of endemic calf morbidity and mortality due to *T. vitulorum,* by provision of the FMB5s and NMBs that eliminated calf mortalities, produced rapid reduction in FEC relative to the control calves and a higher ADG (230 g) than the control calves (170 g). Additional trials corroborated that FMB5 access was associated with higher predicted ADG (200 g) compared to control calves (150 g). The initial TMB5 trial examined *Fasciola spp*. suppression over a 12-week period, with a reduction in FEC (~90%) and increased ADG (201 g) that was superior to the reduction in FEC (~19%), and ADG (124 g) of those with NMB access. A trial with TMB10 observed rapid elimination of the FEC following exposure to this block. Trials with NMB and UMB8 compared the weight gains of young (<8 m. old) calves, grower (>8 m <20 m) cattle and lactating cows, with access to either block enabling impressive ADGs of young calves (298 g, 238 g), growing cattle (214 g, 143 g) and lactating cows (191 g, 179 g), that were vastly superior to baseline un-supplemented free-grazing animals (54–84 g/day). As the trial in the buffalo dairy identified improvements in milk yields of up to 31% from access to UMB10, it was concluded that the superior ADGs in young calves likely reflected the effect of supplementation on lactation yields and that this would inevitably benefit reproductive performance [1,2].

It was noted that the average daily block consumption of the majority of MNBs examined, was variable in the trials although generally in the vicinity of 150 g/day, with the exception that of the trials with UMB8 blocks. In the urea block trials, the consumption of NMBs per animal exceeded that of UMB8s per animal (139–145 g/day versus 116–117 g/day), although it was considered this may have reflected the higher vegetable oil content in UMB8 to limit intake for prevention of urea toxicity risk, with these blocks likely to have been less palatable [1]. This is an observation previously considered, with superior ADGs being determined in cattle accessing NMBs compared with FMB5s [20]. Consumption of the UMB8s, NMBs and EMBs was also less than that described in some literature, with intakes exceeding 200 g/day having commonly been reported [19]. However, this was expected and presumably reflects both the hardness of these high-quality MNBs (following a patented manufacturing process), the type of feed available (e.g., forages versus roughage), and the small stature and mature body weights (146–215 kg) of Lao cattle, with lower metabolisable-energy requirements and feed intakes of Lao indigenous cattle, compared with most other breeds and crossbreed cattle [4,5,6]. Studies of cattle feeding behaviour with digital in-paddock technologies including electronic feeders and walk-over-weighing scales, have demonstrated the effect of forage quantity and quality on the intake of a self-fed supplement MNBs [24,40].

These studies of mostly previously published research conclude that the addition of high-quality MNB or EMB supplementation, is a practical and efficacious large ruminant livestock management strategy in Lao PDR and potentially other developing countries. The technology is capable of significantly improving tropical smallholder livestock production efficiency, in the order of 2.5 to 5 times ADGs over that measured as baseline free-grazing data. Importantly, EMBs have a potential abatement of between 350 to 470 kg CO_2_e. The methodology for GHGe calculations used the IPCC Inventory Software V 2.69 that was developed by the Taskforce on National Greenhouse Gas Inventories. The IPCC National Greenhouse Gas Inventories Programme initiated the development of the new GHG Inventory Software in 2013 with the purpose of implementing Tier1 and Tier2 methodologies in the 2006 IPCC Guidelines for National Greenhouse Gas Inventories. It aims to enable preparation of national GHG inventories in accordance with 2006 IPCC Guidelines, either for complete inventories or for separate categories or groups of categories. The approach to modelling of data reported in this paper was informed using mapping of emissions estimates for ‘other cattle 3.A.1.a.i’ and ‘buffalo 3.A.1.b’ (using the IPCC 2006 updated in 2019 for the most disaggregated level for inventory categories) and the Tier 2 modelling associated with the livestock category in question. The IPCC Inventory Software implements Tier 1 methods for all sectors and Tier 2 methods for Agricultural categories. Briefly, the model is parameterised using feed intake, animal mass, average weight gain from measured data and use animal default values for coefficients for calculating net energy (NE; maintenance, activity, work, pregnancy and growth), digestible energy and gross energy. The IPCC emissions factors for enteric fermentation were used to calculate the total greenhouse gas emissions.

The use of MNBs is suggested as a potentially important project ‘entry point’ intervention for livestock development, motivating farmers to improve their cattle and buffalo production efficiency and increasing receptivity for health and biosecurity co-learning programs [1,2]. Importantly, MNBs address the extended lag period when forage plantations are being established, especially in dry seasons when nutrition is limiting. Further, in developing countries where lack of cattle handling equipment means administration of medication is difficult, high-quality MNBs provide a convenient form of delivering some anthelmintics [20,21]. In addition to anthelmintics, urea and GHGe reducing agents, there has been considerable interest in the use of red seaweed, and in particular *Asparagopsis taxiformis* to increase production of cattle and to reduce GHGe [41,42,43,44]. Several different seaweeds have been fed to cattle and include brown seaweeds *Ascophyllum nodosum,* and *Saragssum wightii,* with a commercial product developed based on A. *nodosum* [43]. A recent meta-analysis on the limited data available on dietary supplementation of cattle with seaweed, indicates a significant and substantial reduction in methane yield despite marked heterogeneity in the results, with one comparison claiming methane yield was reduced by 97%. It was concluded that whilst there was evidence of potential benefits from using seaweed to improve production and reduce methane yields, more in vivo experiments are required, particularly to identify sources of heterogeneity in methane responses occurring in studies on applications and potential risks for seaweed use in bovine diets [44].

A multi-intervention livestock development strategy involving a combination of health and nutritional interventions including provision of high-quality MNBs has now been proposed as a ‘scale-out’ strategy to assist smallholder large ruminant livestock farming efficiency in developing countries [1,45] with potential applications in developed countries [46]. This approach, whilst providing moderate reductions in GHGe, also provides considerable increases in animal performance through improved rumen digestion efficiency. As the approach aligns directly to the aspirations of the Paris Agreement and the Global Methane Pledge [13,15], it is an observation of significance to developing nations as it provides a platform for future investments in sustainable food production that meets several SDGs. The approach of assessing changes in GHGe intensity identified in this paper, confirms that UNFCCC (United Nations Framework Convention on Climate Change) clean development mechanism approaches used in dairy (AMS-BK) could be used for beef production and buffalo [33,34,35,36,37,38]. Further, the use of high-quality MNBs may be a simple motivator for these communities to increase the efficiency of large ruminant production, improving rural livelihoods, food security, and potentially, reducing GHGe from ASFs. It potentially offers important socioeconomic benefits for improved community resilience in poor rural communities, in addition to potentially enabling the global livestock sector to reduce GHGe by as much as 30% and assisting to diminish the risks of the impending climate change catastrophe.

In addition to addressing climate change concerns of large and small ruminant production, renewed attention is required for improved animal welfare in developing livestock systems and have them increasingly aligned with current global animal welfare strategies [47,48,49]. A recent analysis examined the proportion of published articles in livestock research (n = 563) within the period of 2015–2021, that have conducted animal welfare assessments combining objective measures of physiological stress and evaluation of climate change factors in relation to livestock productivity [49]. The review identified that although research into animal welfare assessment, objective measures of stress and climate change has been applied across both monogastric and ruminant livestock production systems, there is a shortfall of investigations on how these key factors interact to influence livestock production and the emerging technologies that can boost the quantitative evaluation of animal welfare in both intensive and extensive production systems.

Our paper and a recently published review [47] draw attention to the reality that in the smallholder livestock farming systems that occur mostly in developing countries, both the concepts and practices of improved animal production, health and welfare have been slow to gain traction in many regions. The rapidly improving economies in the GMS have created a sustained increase in demand for ASFs in the region. This has increased both transboundary disease risks from extended livestock movements, and emerging infectious disease risks from persistence of unhygienic ‘wet markets’ where the sale of wildlife adjacent to livestock is still tolerated. Change management to improve regional biosecurity is urgently required, yet motivating farmers to adopt this is known to be very challenging [7,50]. The use of highly visible interventions capable of creating rapid system change that motivates farmers is required. Access to MNBs to increase productivity, accompanied by targeted health preventive strategies (e.g., vaccination, MNBs) to reduce disease risk, are considered most likely to drive the practice change urgently required. Further, perhaps the more recent recognition that linkage of the SDGs with animal welfare can assist, with opinion that improving animal welfare would contribute positively to the achievement of the SDGs and similarly, achieving the SDGs, would help improve animal welfare [46,49]. Hopefully, there is an awakening occurring in the post-pandemic era that One Health should be a collaborative priority for all medical and veterinary health authorities and that this may create a more receptive environment for the change management required in progressing both animal health and welfare through productivity innovations, assisting GHGe mitigation from the currently inefficient livestock systems, particularly in developing countries.

## 10. Conclusions

Examination of published studies in Lao PDR and other reports on recent developments in nutritional management innovations, confirm that high-quality molasses blocks provide a robust means of delivering a nutritional and health intervention that substantially boosts smallholder large ruminant productivity. The molasses block intervention has the convenience of tailoring the feeding system to address particular needs, including the addition of an anthelmintics for endoparasite control, and/or addition of urea to increase nitrogen availability and improve rumen fermentation of low-quality forages. Importantly, the addition of GHGe reducing agents to MNBs to create EMBs, may assist in reducing the carbon/methane footprint of large ruminant livestock production, potentially contributing abatement of up to 470 kg CO_2_e per block consumed. It is suggested that MNBs are capable of contributing to improved global large ruminant livestock production system efficiencies, assisting the change management urgently required to address global food security, biosecurity deficits, and potentially, ecosystem health through improvements to climate crisis management.

## Data Availability

Not applicable as the majority of the data presented in this review has been published.
